# HIV and Cardiovascular Disease: Contribution of HIV-Infected Macrophages to Development of Atherosclerosis

**DOI:** 10.1371/journal.pmed.0040043

**Published:** 2007-01-30

**Authors:** Michael Bukrinsky, Dmitri Sviridov

In their Perspective published in *PLoS Medicine* [1], Carr and Ory provide a fair review of our recent paper in *PLoS Biology* [2]. However, we believe their commentary puts too much emphasis on the role of low high-density lipoprotein (HDL) cholesterol levels as a cause of atherosclerosis in HIV-infected patients. While HDL cholesterol levels are reduced in untreated HIV infection [3], and defects in reverse cholesterol transport (RCT) that we reported may well be a contributing factor to this abnormality, initiation of antiretroviral therapy restores HDL levels [4]. Although the development of dyslipidemia with prolonged use of anti-HIV drugs again lowers HDL cholesterol and in addition raises very low-density lipoprotein and low-density lipoprotein levels [5], it is unlikely that HIV infection contributes significantly to these effects. Indeed, most HDL comes from the liver and intestine, which are responsible for maintaining plasma HDL levels [6], but neither hepatocytes nor enterocytes are infected by HIV. Also, the number of HIV-infected cells in treated patients is relatively low to account for any general changes in concentration of plasma lipoproteins.

We suggest a different model that provides a simple connection between HIV-induced impairment of the cellular step of RCT and pathogenesis of atherosclerosis. We propose that RCT-defective HIV-infected macrophages contribute to development of atherosclerosis in HIV patients by converting into foam cells and initiating plaque formation in the vessel wall. Indeed, specific inactivation of *ABCA1* in macrophages has been shown to induce atherosclerosis in a mouse model independently from plasma HDL level [7]. Plaque formation through this mechanism can begin even on the background of normal HDL but would be greatly accelerated by dyslipidemia, a condition observed in HIV patients treated with antiretroviral therapy. Given that even fully suppressive HAART (highly active antiretroviral therapy) does not eliminate long-lived productive reservoirs of the virus and that macrophages are a likely potential component of these reservoirs [8], long-lived HIV-infected macrophages may contribute to atherosclerotic plaque formation long after initiation of HAART. Consistent with this hypothesis, it was found that the majority of cardiovascular events in HIV-infected patients are “one plaque” events [9].

Therefore, several lines of evidence indirectly implicate HIV-infected macrophages in the pathogenesis of atherosclerosis in HIV patients. Future studies will determine the role of HIV-induced RCT impairment in this process and are expected to provide an understanding of the connection between HIV infection and atherosclerosis and to identify novel treatment targets for both diseases.
